# Optimization of Pulmonary Vasculature Tridimensional Phenotyping in The Rat Fetus

**DOI:** 10.1038/s41598-018-37906-8

**Published:** 2019-02-04

**Authors:** Emrah Aydin, Brittany Levy, Marc Oria, Hussam Nachabe, Foong-Yen Lim, Jose L. Peiro

**Affiliations:** 1 Center for Fetal and Placental Research, Division of Pediatric General and Thoracic Surgery, Cincinnati Children’s Hospital Medical Center (CCHMC), Ohio, USA; 2 Department of Pediatric Surgery, Koç University School of Medicine, Istanbul, Turkey

## Abstract

Comparative, functional, developmental, and some morphological studies on animal anatomy require accurate visualization of three-dimensional structures. Nowadays, several widely applicable methods exist for non-destructive whole-mount imaging of animal tissues. The purpose of this study was to optimize specimen preparation and develop a method for quantitative analysis of the total pulmonary vasculature in fetal rats. Tissues were harvested at E21 and fetuses fixed overnight in 4% paraformaldehyde/phosphate buffered saline. They were treated with 25% Lugol solution for 72 hours to ensure perfusion. Four different methods were used for fetal specimen preparation; isolated lung, upper torso, direct right ventricle contrast injection, and whole body with partial thoracic skin excision. The microCT scan was performed, and pulmonary vasculature was segmented. Vessels were analyzed for diameter, length, and branching. Of the four preparation methods, only whole body with partial thoracic skin excision resulted in adequate reconstruction of the pulmonary vasculature. In silico generated 3D images gathered by micro CT showed pulmonary vasculature distributed throughout the lung, which was representative of the shape and structure of the lungs. The mean number of vessels segmented in the pulmonary tree was 900 ± 24 with a mean diameter of 134.13 µm (range 40.72–265.69 µm). While up to the 30^th^ generation of vessels could be segmented, both for arteries and veins, the majority of branching was between the 21^st^ and 30^th^ generations. Passive diffusion of contrast material enables quantitative analysis of the fetal pulmonary vasculature. This technique is a useful tool to analyze the characteristics and quantify the fetal pulmonary vasculature.

## Introduction

Vascular networks provide pathways for organ nutrient and waste exchange, providing a scaffolding for cellular growth and organ development. The particular branching pattern of these vascular networks allows for appropriate blood supply to organs and tissues, and specific flow patterns allow for individual processes to occur and promote specialized organ function. Vascular networking within each individual, known as arborization, can vary slightly within the normal range, however, in diseased states, these networks can be significantly altered depending on cellular signals regulating angiogenesis within the organ^1^.

In the lung, the vascular network leads the way for tissue growth and pulmonary development during the embryonic and fetal periods. Later, this network enables oxygenation via alveolar oxygen exchange, which requires a particular vascularization pattern between arterial and venous flow. There are many diseases, either acquired or congenital, that affect vascular wall remodeling and vessel number, such as emphysema, congenital diaphragmatic hernia and bilateral renal agenesis^2,3^. In order to distinguish aberrant vascularization, characterization and comparison of normal fetal vasculature in individuals utilizing simple and reproducible methods is necessary.

Historically, histological evaluation was used to analyze lung tissue^4^. Histological analysis can be done in animal studies to determine vessel distribution; however, this technique requires multiple staining procedures which render the sample unusable for other downstream analysis. Additionally, a detailed and complete analysis of lung vasculature is not easily obtained using histologic evaluation due to a random sampling of individual histologic slices or is extremely labor-intensive using stereology techniques.

Alternatives to histology have previously been attempted, such as corrosion casts, arteriograms and computerized tomography (CT) scans^4–7^. However, these methods generally evaluate around 0.1% of the vasculature in the lung which were not representative of the lung as a whole^4–7^. Furthermore, these methodologies were limited by the method of contrast agent introduction. Intravenous delivery of iodine-based agents via tail vein resulted in unequal distribution of the contrast dye through the lungs. Cast-like contrast agents can cause overfilling of the vessels resulting in inaccurate measurement of vessel diameter, and potentially disrupt tissue structure due to perfusion pressure. *Ex vivo* contrast infusion under pressure causes changes in vessel orientation, demonstrating further limitations and potential errors in these imaging techniques^7^.

Like traditional CT scan imaging, micro CT builds a 3-dimensional image based upon consolidation of multiple x-ray images. The resolution of micro CT for vasculature has been improved providing the ability to precisely define the microvasculature within developing organs^8^. Usage of imaging stains allows for further contrast to better evaluate soft tissue anatomy and vasculature patterns. Multiple stains have been applied to the micro CT technique, the most commonly used including phosphotungstic acid (PTA), iodine potassium iodide (IKI, Lugol’s solution), and 1% iodine in ethanol or methanol^9^. The non-volatile and excellent penetrative properties of these stains allow for detailed imaging of anatomy through micro CT, while still preserving the sample for further experimental0 manipulation and analysis^8^. In light of these developments, the purpose of this study was to develop a new sample preparation method for quantitative analysis of the total fetal pulmonary vasculature in rats, that is easy to reproduce and validate, and has potential to be translated into human studies.

## Methods

### Experimental Design

Following approval of IACUC protocol #2016-0068 by the Cincinnati Children’s Research Foundation Institutional Animal Care and Use Committee, age-matched Sprague Dawley rats were mated. All experiments complied with the National Institutes of Health Guide for the care and use of Laboratory Animals (NIH Publications No. 80023, revised 1978). The date when the vaginal plug was seen was accepted as embryonic day 0 (E0). At embryonic day 21 (E21) (full gestational length 22–23 days), all fetuses were harvested by cesarean section after maternal inhalation anesthesia (isoflurane). Overall, 16 fetuses from two dams were used in the study. As red blood cells are necessary for intravascular Lugol binding, the umbilical cord was tied immediately to prevent blood loss and pups were euthanized by carbon dioxide.

### Specimen Preparation

Lugol solution (10 g KI and 5 g I_2_ in 100 ml H_2_O) was used as an intravascular contrast agent. To allow better diffusion and reduce shrinkage of soft tissue, it was diluted with de-ionized H_2_O to achieve a physiologically isotonic solution^10^. Fetuses were fixed overnight in 4% paraformaldehyde (PFA) within phosphate buffered saline (PBS). They were treated with 25% Lugol solution for 72 hours. To compare visualization of the pulmonary tree, additional distal vascular branches from four different specimen groups were prepared, including isolated lung, upper half of torso, direct right ventricle injection (injection group), and whole body with partial excision of the thoracic skin (2 mm in diameter) since the skin is the primary barrier preventing Lugol perfusion. Four fetuses in each group were individually imaged to assess the diffusion of iodine into the pulmonary vasculature and to determine which preparation resulted in optimal Lugol penetration.

### Micro CT Imaging

The micro CT scanner used in this study was a MicroCAT II (ImTek, Inc., Knoxville, TN). For the scanner geometry employed here, the usable field of view was slightly less than 3 × 2 cm. Raw data was acquired over a 55-minute scan using the following parameters: 80 kVp, 200 µA, 800 projections, 0.25° increment, 3.3 s exposure, with 2-by-2 binning of the 3072 × 2048 element CCD detector. The projection data was corrected for distortion and detector anomalies and then reconstructed by Feldkamp cone beam filtered back projection, resulting in a 3D image with an isotropic voxel size of 19 µm (isotropic resolution of 21 µm, calculated by the Imtek Resolution Estimator software, based on scanner geometry, source, and camera conditions).

### Image analysis

Commercial software, Analyze 12.0 (Rochester, MN), was used for data acquisition and 3D reconstruction of the pulmonary arterial tree from the data generated by micro CT. A 3D reconstruction of the pulmonary vascular tree was constructed using multiple steps. First, the area of interest was selected manually and then a threshold segmentation was optimized for the specimen. Seeds, which were defined as one of the pixels in the area of interest, were selected on the stained vasculature and the object was extracted by the ‘region grow’ function. Vessels other than those belonging to the pulmonary system were excluded by the ‘object separator’ function which can be manually adjusted by the user. The high-resolution 3D renderings of the vascular tree were generated using a 3D volume rendering technique. Tree analysis, i.e. quantification of diameter and length of the pulmonary vessels, was done using the ‘generate tree’ function^11,12^. Lastly, the number of branches and generations were calculated automatically by the software.

### Statistical analysis

Statistical analysis was performed with IBM SPSS Statistics 20.0.0 (Chicago, IL). Data are expressed as mean ± standard deviation. Kolmogorov-Smirnov test was used to demonstrate normal distribution. One-Way ANOVA was used for homogeneity of the variables. Student’s T-test and Pearson correlation were used for parametric data. Statistical associations were considered significant if the p-value was < 0.05.

## Results

### Contrast Distribution

**Figure 1:**
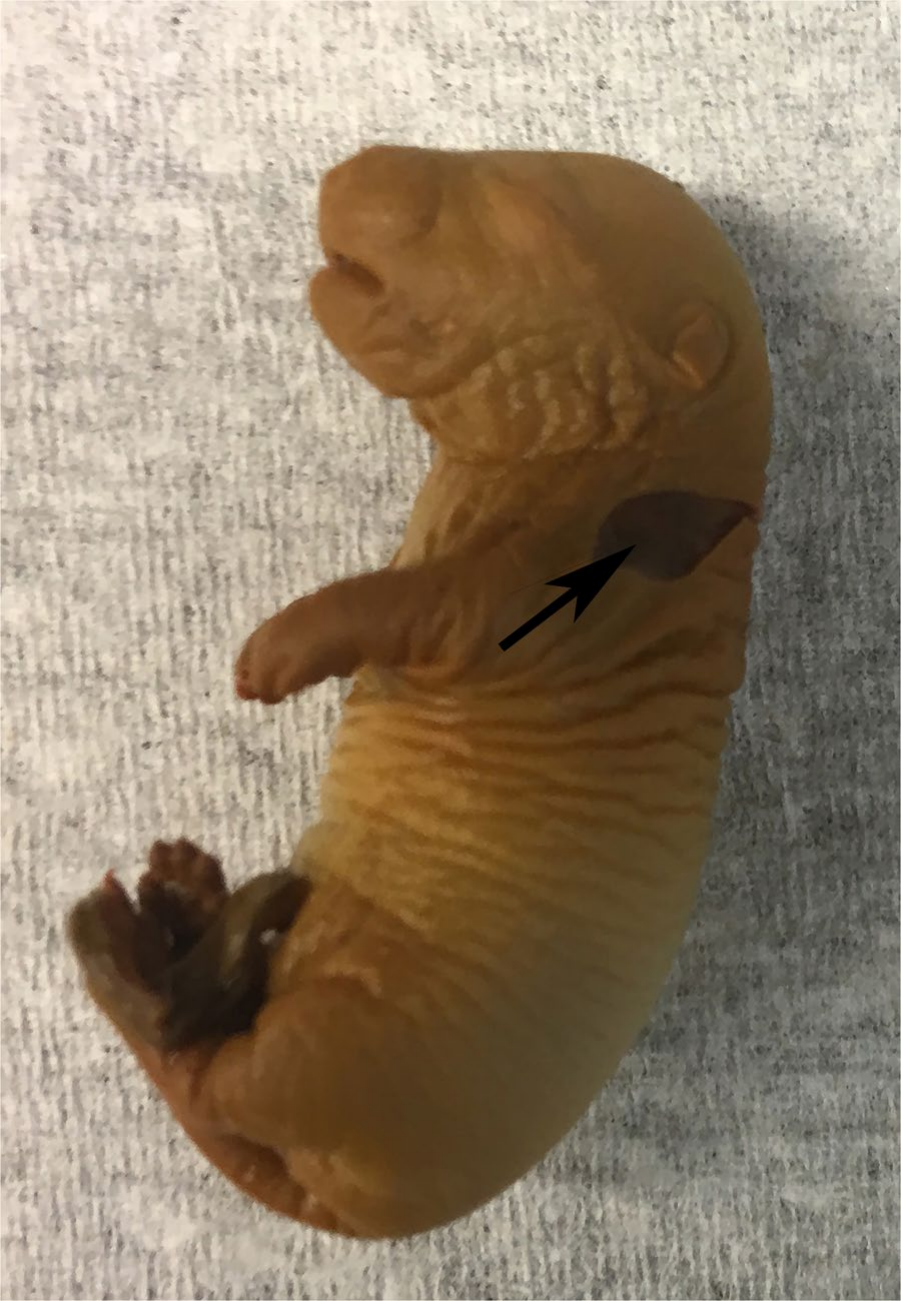
Partially peeled skin of a rat fetus (arrow).

**Figure 2:**
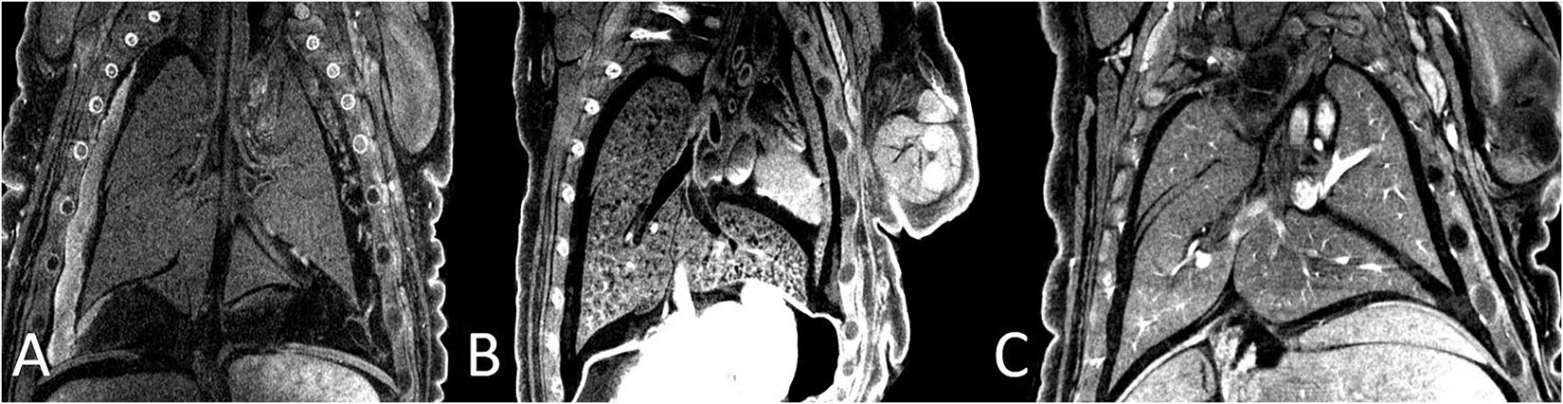
Micro CT images of the fetuses. (**A**) Injection of the contrast directly into the right ventricle; (**B**) Upper half body of the fetus embedded into the Lugol solution; (**C**) Partial peeling of the skin embedded in Lugol solution.

Different methods to introduce the iodine were tried to improve the adequacy of iodine stain penetration into the pulmonary tree. In the pups with partially excised skin (Fig. 1), Lugol diffused homogenously throughout the vessels providing detailed imaging of the lung vasculature (Fig. 2). On the other hand, isolated whole lung from the fixed fetus, exposed to Lugol solution yielded no visible vessels on the CT scan. Direct injection of the contrast material into the right ventricle, on the other hand, was an unexpected failure, with almost none of the vasculature visible and significant contrast agent pooled in the heart. When the fetal torso was placed in the Lugol solution, there was only partial penetration of the iodine stain due to excessive blood loss, and the complete pulmonary tree could not be displayed.

### Visualization of fetal lung microvasculature

**Figure 3:**
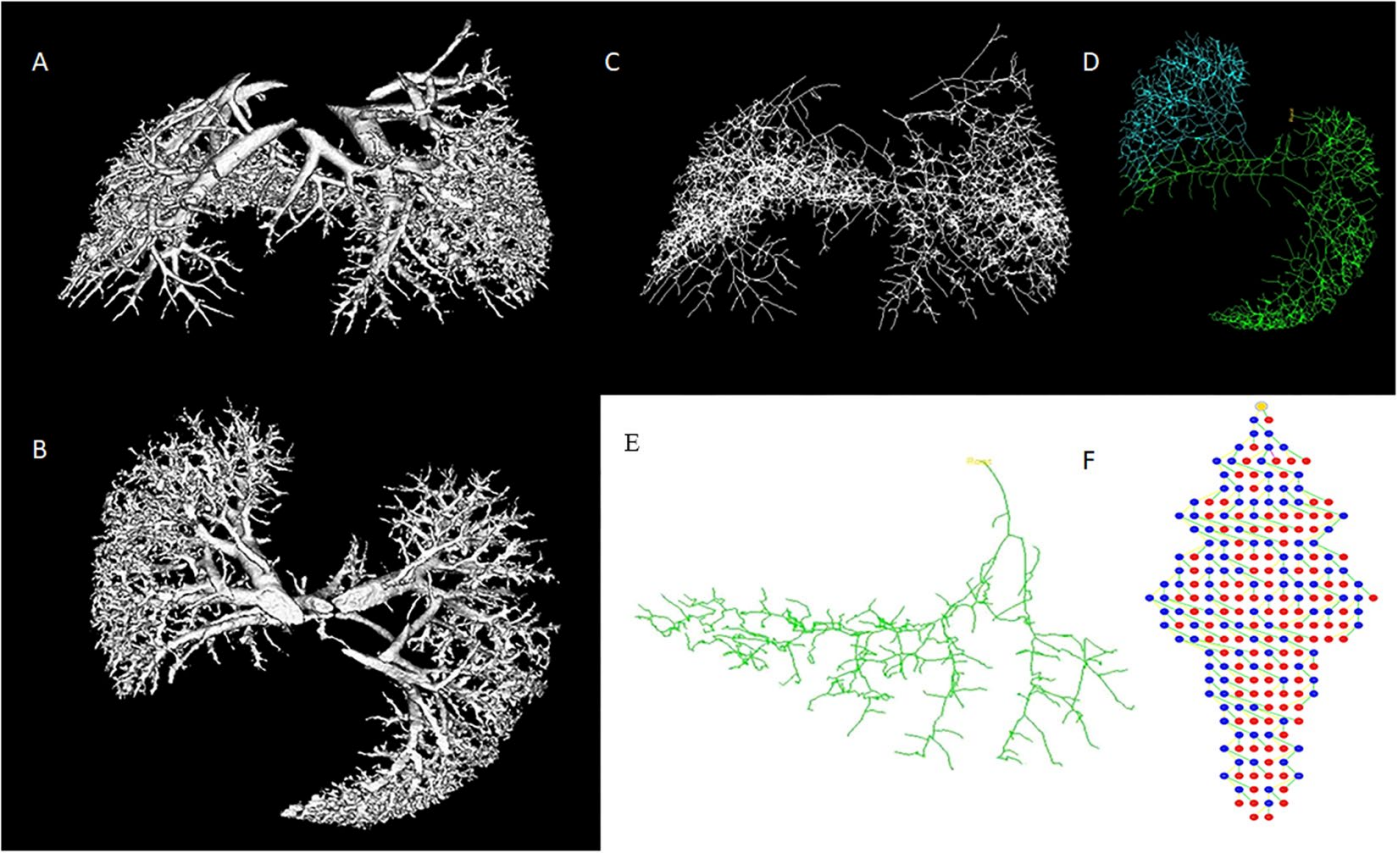
Three-dimensional images of the entire lung vasculature by micro CT scan after *ex-vivo* Lugol application on partially excised skin specimen on coronal and axial planes (**A**,**B**), Skeletonization of the entire lung vasculature (**C**), and colored image that allows the analysis of vasculature of a single lobe (**D**), Skeleton of one network of vessels in the right lobe (**E**) with mapping which helps measure all parameters of the vessels either selecting the vessel junctions (blue [mid-branches]and red [end branches]) or the vessels themselves (yellow and green) (**F**).

Four rat fetuses were scanned in each group. The partially excised skin technique in which there was not any significant blood loss, was the only successful preparation that resulted in the visualization of the microarchitecture in all 4 samples. This technique revealed both the side branches of the high generation vessels and 3D interconnectivity of the whole fetal lung. Per iodine stains, only the blood, any bleeding in pups impaired filling of a whole pulmonary vascular tree down to the level of alveolar capillaries and a clear visualization of the pulmonary microcirculation could not be achieved. The 3D images gathered by micro CT and processed by the software showed pulmonary arteries, pulmonary veins, and micro vessels distributed through the lung on E21 of a rat fetus (Fig. 3). The pulmonary vascular tree was representative of the shape and structure of the lungs.

### Morphometry

**Figure 4:**
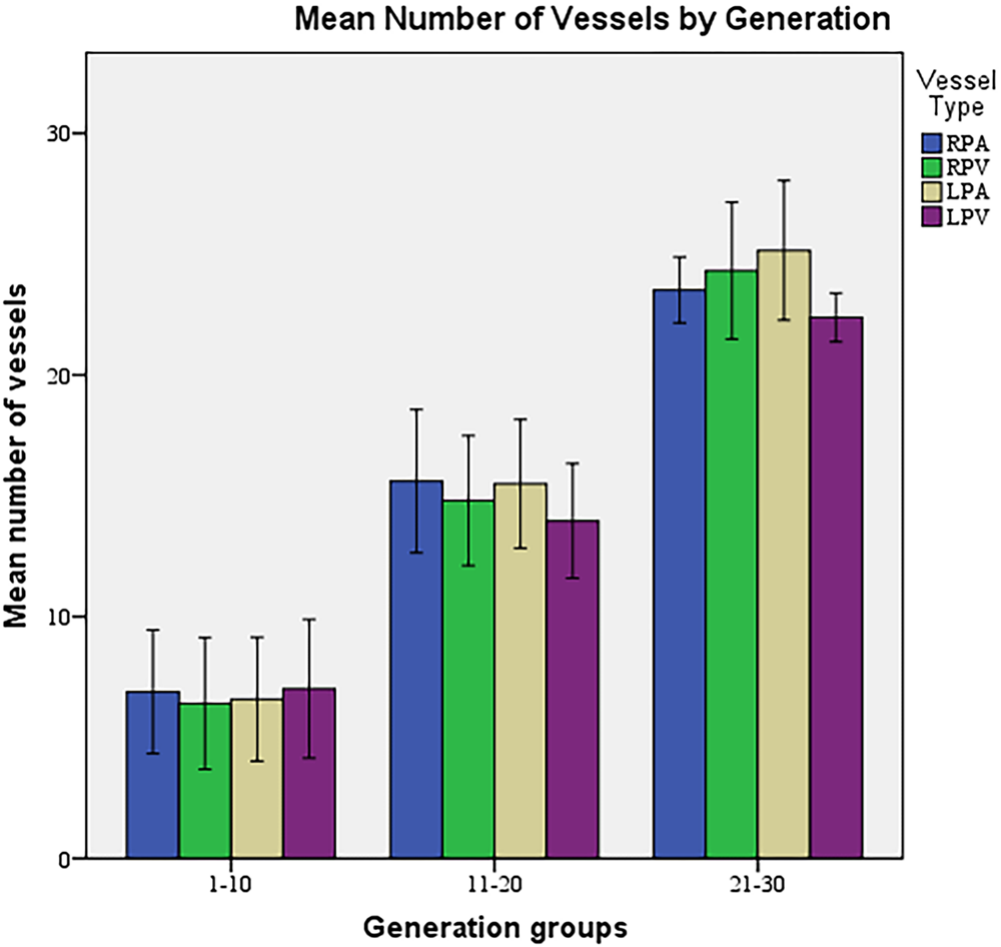
The mean vessel number of each vessel type across generation groups. (RPA: Right pulmonary artery, RPV: Right pulmonary vein, LPA: Left pulmonary artery, LPV: Left pulmonary vein.).

The mean number of vessels segmented in the pulmonary tree was 900 ± 24 (mean ± SD) with a mean diameter of 134.13 ± 53.00 µm (range 40.72–265.69 µm). Comparison of the measurements between arteries and veins are summarized in Table 1. While up to the 30^th^ generation of vessels were able to be segmented both in arteries and veins, the majority of branches were between the 11^th^ and 30^th^ generations (Fig. 4). The comparison of the number of vessels (arteries vs veins on the same lung) did not reveal any statistical significance (p > 0.005).

**Table 1:** The morphometric results per vessel type.

	RPA	RPV	LPA	LPV
Area of vessel (µm^2^)	36.38 ± 28.86	52.46 ± 42.01	40.64 ± 28.03	47.01 ± 39.16
Vessel length (µm)	21.02 ± 14.31	20.64 ± 14.4	19.3 ± 13.04	22.26 ± 13.51
Diameter of the vessel (µm)	122.97 ± 46.07	145.78 ± 60.18	131.14 ± 45.46	137.68 ± 57.72
Circumference of the vessel (µm)	386.32 ± 144.75	457.98 ± 189.05	411.97 ± 142.82	432.53 ± 181.34
L/D ratio	0.20 ± 0.15	0.18 ± 0.14	0.18 ± 0.14	0.21 ± 0.16

(RPA: Right pulmonary artery, RPV: Right pulmonary vein, LPA: Left pulmonary artery, LPV: Left pulmonary vein, L/D ratio: Vessel length to diameter ratio.) (Values expressed as means ± standard deviations).

## Discussion

The application of fetal lung vasculature modeling spans both fundamental embryology as well as an understanding of congenital lung disease. Congenital lung diseases such as pulmonary hypertension and congenital diaphragmatic hernia cause significant morbidity and mortality during the neonatal period in humans^13,14^. Further understanding of both quantitative and comparative analysis of fetal pulmonary tree development and maturation is necessary to address many questions regarding these pathologies. This study provides a foundation to identify and compare normal lung vasculature variation as well as the fetal vasculature in the diseased organ to fully investigate etiology and pathology.

The method reported in this study combines the precise imaging capabilities of micro CT reconstruction while avoiding many of the limitations related to the introduction of contrast agents^9,15^. Passive diffusion of Lugol prevents overinflation of the vessels, previously encountered by infusion of the contrast with controlled pressure, or unequal distribution of the contrast material throughout the lungs. This methodology of passive perfusion maintains similar dimensions in the lungs as the postnatal physiological ones, reducing concern for changes in vessel orientation. The skin proved to be the most preventative barrier for Lugol diffusion, therefore partial excision of the skin allowed adequate perfusion of the vasculature^10^. Since the red blood cells are necessary for Lugol intravascular binding, prevention of bleeding is crucial, as it is the main cause of unequal distribution of the contrast in complete removal of the skin. Loss of blood from the samples prior to fixation, as well as Lugol diffusion, caused reduced image quality due to the non-homogenous distribution of the contrast agent.

The micro CT technique, with the introduction of a Lugol-based contrast agent by passive diffusion, has multiple advantages compared to previous techniques mentioned in the literature^1,6,7^. Aided by semi-automated, commercially available software, it requires less processing and provides the ability to image a whole specimen. Due to the nature of Lugol, the introduction of contrast and subsequent imaging does not cause sample destruction, allowing further opportunity for the same samples to be evaluated by different methods. The scan time of a micro CT is about 55 minutes, with post-scan processing of 15 minutes, significantly reducing time to obtain morphometry data. The resolution of micro CT for vasculature has significantly improved and provides the ability to precisely define the microvasculature within developing organs^8^. Although this study focused on lung development, with proper staining and preparation techniques, vasculature patterns can be visualized using micro CT in any vascular organ throughout fetal development. Furthermore, this early analysis of lung anatomical growth has application for early detection of abnormalities in embryologic and fetal development. The gross and morphometric findings in this study are comparable with the studies published in the literature with other methods^1,7,12^. As seen in previous studies, this technique also allows us to segment up to the 30^th^ generation of the vessels^1,7,12^. This technique, with the aid of the automated software, helps to analyze the lung either as a whole, by sides, or by individual lobes.

Unfortunately other than being an *ex-vivo* technique, micro CT has its own described limitations, particularly in the design and set up of the experiment and system^9^. Efforts have been made to translate these studies into *in-vivo* studies with MRI in order to prevent radiation exposure. As with traditional imaging modalities, the quality of the image is dependent on the minimization of artifact from incorrect positioning or interference. Proper geometric dimensions must be adhered to for proper 3-dimensional reconstruction^9^. This could be achieved by fixing the specimens on to the scanner with the use of pins. Tissue shrinkage caused by both PFA 4% and I_2_KI is a limiting factor, which could alter the architecture of the tissue. The shrinkage effect of the I_2_KI is mostly concentration dependent as reported by Vickerton *et al*., in order to prevent this, we utilized the previously optimized concentration^15^. However, micro CT provides the ideal imaging modality to identify specific branching patterns and for comparison of these patterns across fetal samples. Determination of organ-specific vascularization patterns and modeling of these vascular trees is imperative for further identification of disease impact on embryological and developmental status.

Lung vasculature is of particular interest in the embryo and fetal development due to the significant impact of the pulmonary vascular disease in the neonatal period. Previous investigation has utilized micro CT to better visualize lung vasculature and identify vascular patterning over multiple ages^7^. We demonstrate the feasibility of the micro CT usage with the help of a commercial software in the prenatal period. While our current study presents only micro CT data, we are developing MRI validation, and this may allow us to translate this technique into human samples in the future. However, further analysis of lung vasculature distribution should be investigated in disease models throughout gestation to determine the implications of arterial networking in neonatal pulmonary disease.

## References

[1] Yang J, Yu LX, Rennie MY, Sled JG, Henkelman RM (2010). Comparative structural and hemodynamic analysis of vascular trees. AJP - Hear Circ Physiol..

[2] Voelkel N. F., Douglas I. S. & Nicolls M. Angiogenesis in chronic lung disease. *Chest*. **131**(3), 10.1378/chest.06-2453 (2007). 10.1378/chest.06-2453 PMC4396181 17356107

[3] Kool H, Mous D, Tibboel D, de Klein A, Rottier RJ (2014). Pulmonary vascular development goes awry in congenital lung abnormalities. Birth Defects Res Part C - Embryo Today Rev..

[4] Hsia CCW, Hyde DM, Ochs M, Weibel ER (2010). How to measure lung structure-What for? On the “Standards for the Quantitative Assessment of Lung Structure”. Respir Physiol Neurobiol..

[5] Hsia CCW, Hyde DM, Ochs M, Weibel ER (2010). An official research policy statement of the American Thoracic Society/European Respiratory Society: Standards for quantitative assessment of lung structure. Am J Respir Crit Care Med..

[6] Dunnill MS (1962). Quantitative Methods in the Study of Pulmonary Pathology. Thorax..

[7] Phillips MR (2017). A method for evaluating the murine pulmonary vasculature using micro-computed tomography. J Surg Res..

[8] Powell KA, Wilson D (2012). 3-Dimensional Imaging Modalities for Phenotyping Genetically Engineered Mice. Vet Pathol..

[9] Bentley MD, Ortiz MC, Ritman EL, Romero JC (2002). The use of microcomputed tomography to study microvasculature in small rodents. Am J Physiol Regul Integr Comp Physiol..

[10] Degenhardt K, Wright AC, Horng D, Padmanabhan A, Epstein JA (2010). Rapid 3D phenotyping of cardiovascular development in mouse embryos by micro-CT with iodine staining. Circ Cardiovasc Imaging..

[11] Tran S. & Shih L. Efficient 3D binary image skeletonization Efficient 3D binary image skeletonization. C*omput Syst Bioinforma Conf 2005 Work Poster Abstr IEEE*. 364–372, 10.1109/CSBW.2005.57 (2005).

[12] Counter WB, Wang IQ, Farncombe TH, Labiris NR (2013). Airway and pulmonary vascular measurements using contrast-enhanced micro-CT in rodents. Am J Physiol Lung Cell Mol Physiol..

[13] Congenital T, Hernia D, Group S (2007). Defect Size Determines Survival in Infants With Congenital Diaphragmatic Hernia. Pediatrics..

[14] Lazar DA (2011). Impact of prenatal evaluation and protocol-based perinatal management on congenital diaphragmatic hernia outcomes. In: Journal of Pediatric Surgery..

[15] Vickerton P, Jarvis J, Jeffery N (2013). Concentration-dependent specimen shrinkage in iodine-enhanced microCT. J Anat..

